# Case in Which Prune Juice and Orange-Colored Sputa Were Present without Pneumonia

**Published:** 1852-10

**Authors:** J. Wyatt Crane

**Affiliations:** M. D., Edinburg, Licentiate of the Royal College of Physicians, &c.


### PATHOLOGY AND PRACTICE OF MEDICINE.

*Case in which Prune Juice and Orange-Coloured Sputa were present without Pneumonia.* By J. Wyatt Crane, M. D., Edinburg, Licentiate of the Royal College of Physicians, &c.—No opinion in our profession has been so long and so universally received as, that the rustiness, or orange color of the sputa is, when present, pathognomonic of the existence of pneumonia. Valleix, one of the most guarded and accurate, as well as the most recent of systematic writers, says, p. 420, vol. 1, 2nd edit. :—" Ces crachats excepté ceux qui sont colorès en vert sont pathognomoniques de la pneumonie. Leur absence, il est vrai, ne prouve pas absolument que cette maladie n'existe pas, mais leur presence en constate l'existence.' I have, however, had a case under my care lately, where these colorations of the sputa have been present on several occasions, and in which, I am satisfied, both from the absence of the general and physical symptoms of pneumonia, and from the frequency of the occurrence of the disease, its march, and its duration, that pneumonia did not exist. I trust, therefore, that the case may be considered of sufficient interest to prove my apology for laying it before your readers; and, as I conceive that the cause of the coloration of the sputa must be sought for in a pre-existing state of disease, it will be necessary for me to detail somewhat more minutely than might otherwise be necessary, the state of the patient above a year previously to their occurrence, at which epoch I was consulted, in consequence of his finding that he could not walk a short distance without experiencing a feeling of distension and oppression at the præcordia, which compelled him to stop suddenly; and, as these symptoms were likewise accompanied by a loss of muscular power in the lower extremities, rendering it impossible for him to proceed, he was obliged to lay hold of the nearest object to prevent himself from falling; he described himself as suffering from no palpitation, inconvenience, or difficulty of progression, until the moment when the seizure occurred, which it did when he had walked about fifty yards, and that in a few minutes, the oppression, &c., disappeared, until their sudden recurrence when he had walked about fifty yards further. His age was eighty-seven; notwithstanding, his bodily and mental powers were but little impaired, and were remarkably vigorous for his time of life; his appetite and digestion were good; he did not complain of palpitation of the heart, or pain anywhere, and when at rest, felt pretty well; the only complaint he made being a slight feeling of tightness at the epigastrium, of insufficient sleep, and occasional noises in the head, with a slight cough and spitting, principally in the morning; his general health had always been perfect. The pulse was 65, small, and intermittent, generally to the extent of about two intermissions in the minute; on percussion, I found the præcordial dulness a little extended, whilst, in all other parts of the chest, the sound was perfectly normal; on auscultation, an occasional mucous rattle was perceptible, the impulse of the heart was not increased, but a double bruit de soufflet, most clear in the region of the heart, and under the right clavicle, was perceptible in all parts of the chest I inferred, from the above symptoms, the existence of an affection of the valves of the heart, as well as a slight chronic bronchitis, and contented myself with absolutely prohibiting all muscular exertion, or violent emotions; enjoining the patient to take no exercise except in his carriage, and regulating the diet and alvine functions. With these precautions, which he strictly observed, no increase of the symptoms complained of took place, and there was no development of fresh ones, till my attendance was again requested in the middle of the night of the 10th October, 1851, when I found him laboring under the greatest oppression, with a loud wheezing and rattle perceptible at a distance from the bed on which he was lying, but from which he was almost immediately obliged to rise, and sit upright on a chair, in consequence of a sense of impending suffocation. The respiration was exceedingly accelerated and laborious, there was considerable fever, and the pulse was 120. He complained of no pain, but constant cough and copious expectoration of a thin, glairy, and frothy fluid, which in the course of two hours from his first seizure amounted to about a breakfast-cup full; and at this epoch began to assume a rusty color, and to become thicker and more scanty. An attentive examination of the chest detected no other abnormal sounds than the usual bruit de soufflet, almost masked by mucous and sonorous rattles. He had gone to bed perfectly well, and in about two hours afterwards had experienced a difficulty of breathing and accelerated respiration, which he termed "a hurry;" this was succeeded by fever and rapidity of pulse, and this again in a short time by the wheezing and rattling in the chest—by the cough and frequent expectoration before specified. The symptoms were now at their acme, and the dyspnœa was so urgent, that he repeatedly stated his conviction that, unless he were relieved immediately, he would be suffocated. I prescribed two grains of tart. antim., to be taken in divided doses till vomiting ensued; after which he became so much easier that I was enabled to leave him. On visiting him four hours afterwards, at 10 a. m., in conjunction with a French physician who had been requested to meet me, I found him, to my great surprise, suffering from little dyspnœa, no fever, pulse only 80, with scarcely any cough. The sputa which had been expectorated into another cup were scanty, thick, viscid, and deeply colored, "jus de pruneaux," contrasting most remarkably with the contents of their first cup in their quantity, consistency, and color. A minute examination of the chest detected no râle crepitant or dulness on percussion, and nothing abnormal, but a slight and occasional râle muqueux. Heart sounds as usual. The tartrate of antimony was ordered to be continued, and a blister applied. At our visit in the evening, the general symptoms were so much relieved, that but for the effects of the tartrate of antimony, he would have felt himself in his usual state of health. The sputa, however, were still deeply and universally colored "jus de pruneaux," and were very viscid. He passed a pretty good night, and the next day, 11th October, felt himself pretty well, pulse 76, skin cool, no dyspnœa, chest sounds as before, sputa still colored, but the quantity diminished. To continue the antimony, which, I should have stated, had not excited vomiting after the first morning. On the 12th there was no change to note, except a diminution in the pulse to 70 ; sputa as before. On the 13th, 14th, and 15th, the coloration of the sputa, which was the only morbid symptom remaining, gradually diminished, until they became of the character of the usual morning expectoration,—yellowish, white, and translucent; but still so viscid that, on turning the cup upside down, they remained attached to the bottom, and at this period his health was completely restored to its usual state.

On the 20th of November I was again sent for, and found him laboring under an attack of precisely similar character to the previous one, having commenced in the night, in the same manner, and with the same succession in the order of the symptoms, attended, for about two hours, by frequent expectoration of the same copious thin, glairy, frothy fluid, becoming colored, as before, just as it passed into the thicker viscid and deeply-tinged prune-juice sputa; at which period, the dyspnœa fever began to diminish, and continued rapidly to do so, till, in about six hours, the general symptoms had almost completely disappeared ; and, in four days more, the sputa had again regained their natural appearance. The treatment was the same as before, except that no blister was applied. The chest-sounds were as before; and no dulness, on percussion, or crepitous rattle, was preceptible at any time. Similar attacks occurred on the 5th December, 15th December, 29th December, 29th January, 13th February, 6th March, and 12th April; a few days after which the patient left Avranches to return to Ireland. They were of a similar character to the preceding ones, except that the sputa were, in all of them, of an orange color, instead of the dark *jus de pruneaux,* which was only present in the two first; and each successive attack, also was milder than the preceding one, not only with reference to the general symptoms, but also with respect to the coloration of the thick sputa, which gradually became less intense and of shorter duration.

As I have, I fear, already trespassed too much upon your valuable space, I shall only further state, very shortly, that on reflecting upon the peculiarities of the case, I considered that the symptoms were explicable on the supposition that the impediment to the circulation through the lungs, owing to the affection of the valves of the heart, gradually produced distention and obstruction of the capillaries short of the degree which would terminate in pulmonary apoplexy, and that this congestion was relieved by transudation of the serum of the blood; thus accounting for the dyspnœa, etc., and for the first copious thin, frothy expectoration, as well as for the rapid melioration of the oppression and fever, in proportion as the expectoration proceeded; and that the subsequeut coloration of the sputa was due to the more gradual mixture of the coloring particles of the blood with the mucus which was expectorated daily in the chronic bronchitis that had existed previouly —*London Medical Times, September* 4, 1852.

